# Supplementary material for: Modeling glioblastoma heterogeneity as a dynamic network of cell states
Source: Mol Syst Biol. 2021 Sep 16;17(9):e10105. doi: 10.15252/msb.202010105 (PMC8444284; doi:10.15252/msb.202010105)
Supplement: Supplementary file 5 — Source Data for Figure 3 [file MSB-17-e10105-s001.zip › Figure3A_sourcedata/GSEA_3065/nosigdb_state2.GseaPreranked.1622718419215/DOROSZKO_U3054MG_CLUSTER_2.html]

Details for gene set DOROSZKO\_U3054MG\_CLUSTER\_2[GSEA]

|  || Dataset | state2 |
| Phenotype | NoPhenotypeAvailable |
| Upregulated in class | na\_neg |
| GeneSet | DOROSZKO\_U3054MG\_CLUSTER\_2 |
| Enrichment Score (ES) | -0.62609637 |
| Normalized Enrichment Score (NES) | -2.7246966 |
| Nominal p-value | 0.0 |
| FDR q-value | 0.0 |
| FWER p-Value | 0.0 |
Table: GSEA Results Summary

  

Fig 1: Enrichment plot: DOROSZKO\_U3054MG\_CLUSTER\_2      
 Profile of the Running ES Score & Positions of GeneSet Members on the Rank Ordered List

  

| PROBE | GENE SYMBOL | GENE\_TITLE | RANK IN GENE LIST | RANK METRIC SCORE | RUNNING ES | CORE ENRICHMENT || 1 | CCND2 |  |  | 227 | 0.263 | -0.0127 | No |
| 2 | CTDSPL |  |  | 587 | 0.165 | -0.0442 | No |
| 3 | RFLNB |  |  | 595 | 0.164 | -0.0375 | No |
| 4 | TGIF1 |  |  | 1150 | 0.108 | -0.0928 | No |
| 5 | EPB41L2 |  |  | 1200 | 0.105 | -0.0933 | No |
| 6 | STX8 |  |  | 1295 | 0.098 | -0.0991 | No |
| 7 | FARP1 |  |  | 1441 | 0.090 | -0.1108 | No |
| 8 | SOX9 |  |  | 1466 | 0.088 | -0.1094 | No |
| 9 | NAB1 |  |  | 1476 | 0.088 | -0.1063 | No |
| 10 | SPRY1 |  |  | 1623 | 0.081 | -0.1185 | No |
| 11 | SSBP4 |  |  | 1662 | 0.079 | -0.1191 | No |
| 12 | YBX3 |  |  | 1677 | 0.078 | -0.1171 | No |
| 13 | ETV4 |  |  | 1840 | 0.071 | -0.1314 | No |
| 14 | ZNF331 |  |  | 1943 | 0.067 | -0.1395 | No |
| 15 | TMEM98 |  |  | 1991 | 0.065 | -0.1416 | No |
| 16 | ZEB1 |  |  | 1995 | 0.065 | -0.1390 | No |
| 17 | MEOX2 |  |  | 2062 | 0.062 | -0.1434 | No |
| 18 | TMEM167A |  |  | 2099 | 0.061 | -0.1445 | No |
| 19 | CDK6 |  |  | 2124 | 0.060 | -0.1444 | No |
| 20 | SMAD5 |  |  | 2156 | 0.059 | -0.1451 | No |
| 21 | ARHGAP10 |  |  | 2253 | 0.055 | -0.1530 | No |
| 22 | FAM89B |  |  | 2389 | 0.051 | -0.1653 | No |
| 23 | SHOX2 |  |  | 2461 | 0.049 | -0.1708 | No |
| 24 | PYCR1 |  |  | 2522 | 0.047 | -0.1752 | No |
| 25 | PDS5B |  |  | 2565 | 0.046 | -0.1777 | No |
| 26 | LY6E |  |  | 2588 | 0.045 | -0.1780 | No |
| 27 | SPRY4 |  |  | 2656 | 0.043 | -0.1834 | No |
| 28 | PDLIM7 |  |  | 3015 | 0.033 | -0.2207 | No |
| 29 | SDCBP |  |  | 3058 | 0.033 | -0.2238 | No |
| 30 | DAAM1 |  |  | 3107 | 0.031 | -0.2276 | No |
| 31 | CDR2 |  |  | 3139 | 0.031 | -0.2296 | No |
| 32 | FHL3 |  |  | 3201 | 0.030 | -0.2348 | No |
| 33 | CTTNBP2 |  |  | 3302 | 0.028 | -0.2445 | No |
| 34 | ARL1 |  |  | 3309 | 0.027 | -0.2439 | No |
| 35 | RASSF2 |  |  | 3560 | 0.023 | -0.2700 | No |
| 36 | RAMP2 |  |  | 3617 | 0.022 | -0.2751 | No |
| 37 | GLI2 |  |  | 3744 | 0.020 | -0.2879 | No |
| 38 | PDLIM2 |  |  | 3807 | 0.019 | -0.2937 | No |
| 39 | GLIS2 |  |  | 3808 | 0.019 | -0.2929 | No |
| 40 | ANXA11 |  |  | 4466 | 0.008 | -0.3639 | No |
| 41 | SPRED2 |  |  | 4630 | 0.005 | -0.3814 | No |
| 42 | DUSP1 |  |  | 4653 | 0.005 | -0.3835 | No |
| 43 | AFDN |  |  | 4707 | 0.004 | -0.3891 | No |
| 44 | VEGFA |  |  | 4721 | 0.004 | -0.3904 | No |
| 45 | CLEC11A |  |  | 4738 | 0.004 | -0.3919 | No |
| 46 | OLFM2 |  |  | 4808 | 0.003 | -0.3993 | No |
| 47 | CAPN5 |  |  | 4827 | 0.002 | -0.4012 | No |
| 48 | TPPP3 |  |  | 4829 | 0.002 | -0.4012 | No |
| 49 | SAV1 |  |  | 4846 | 0.002 | -0.4028 | No |
| 50 | PTPN9 |  |  | 4863 | 0.002 | -0.4045 | No |
| 51 | C16orf45 |  |  | 5003 | -0.000 | -0.4196 | No |
| 52 | PAWR |  |  | 5100 | -0.002 | -0.4299 | No |
| 53 | S1PR3 |  |  | 5237 | -0.004 | -0.4445 | No |
| 54 | BNIP3L |  |  | 5447 | -0.008 | -0.4669 | No |
| 55 | FAM114A1 |  |  | 5495 | -0.008 | -0.4716 | No |
| 56 | CHN1 |  |  | 5606 | -0.010 | -0.4831 | No |
| 57 | PALLD |  |  | 5615 | -0.010 | -0.4835 | No |
| 58 | PMAIP1 |  |  | 5617 | -0.010 | -0.4831 | No |
| 59 | HCFC1R1 |  |  | 5621 | -0.010 | -0.4830 | No |
| 60 | TSHZ3 |  |  | 5652 | -0.011 | -0.4857 | No |
| 61 | CLMP |  |  | 5717 | -0.012 | -0.4922 | No |
| 62 | GAD1 |  |  | 5744 | -0.012 | -0.4944 | No |
| 63 | ANKH |  |  | 5757 | -0.012 | -0.4952 | No |
| 64 | KLHL13 |  |  | 5792 | -0.013 | -0.4983 | No |
| 65 | S1PR2 |  |  | 5859 | -0.014 | -0.5048 | No |
| 66 | SSPN |  |  | 5862 | -0.014 | -0.5044 | No |
| 67 | TLE2 |  |  | 5909 | -0.015 | -0.5087 | No |
| 68 | EPB41L1 |  |  | 5977 | -0.016 | -0.5152 | No |
| 69 | EFNB1 |  |  | 5978 | -0.016 | -0.5145 | No |
| 70 | MAPK8IP1 |  |  | 6008 | -0.017 | -0.5169 | No |
| 71 | TLE1 |  |  | 6017 | -0.017 | -0.5170 | No |
| 72 | ADAM22 |  |  | 6023 | -0.017 | -0.5168 | No |
| 73 | TWIST1 |  |  | 6072 | -0.018 | -0.5212 | No |
| 74 | ITGB5 |  |  | 6093 | -0.018 | -0.5225 | No |
| 75 | GPX8 |  |  | 6123 | -0.019 | -0.5248 | No |
| 76 | P3H3 |  |  | 6204 | -0.020 | -0.5326 | No |
| 77 | ZDBF2 |  |  | 6252 | -0.021 | -0.5368 | No |
| 78 | LDOC1 |  |  | 6287 | -0.021 | -0.5395 | No |
| 79 | CYTOR |  |  | 6306 | -0.022 | -0.5404 | No |
| 80 | ERRFI1 |  |  | 6445 | -0.024 | -0.5543 | No |
| 81 | NES |  |  | 6455 | -0.025 | -0.5542 | No |
| 82 | PLK3 |  |  | 6607 | -0.027 | -0.5693 | No |
| 83 | LTBP4 |  |  | 6665 | -0.029 | -0.5742 | No |
| 84 | TLN2 |  |  | 6682 | -0.029 | -0.5746 | No |
| 85 | CHSY1 |  |  | 6683 | -0.029 | -0.5733 | No |
| 86 | GLCE |  |  | 6779 | -0.031 | -0.5822 | No |
| 87 | NR3C1 |  |  | 6802 | -0.032 | -0.5832 | No |
| 88 | EFEMP2 |  |  | 6895 | -0.034 | -0.5916 | No |
| 89 | JAM2 |  |  | 6939 | -0.035 | -0.5947 | No |
| 90 | TIAM1 |  |  | 6948 | -0.035 | -0.5940 | No |
| 91 | GPR162 |  |  | 7033 | -0.037 | -0.6014 | No |
| 92 | PEAK1 |  |  | 7039 | -0.038 | -0.6002 | No |
| 93 | SIPA1L1 |  |  | 7046 | -0.038 | -0.5992 | No |
| 94 | NGFR |  |  | 7085 | -0.039 | -0.6015 | No |
| 95 | C1orf54 |  |  | 7100 | -0.039 | -0.6013 | No |
| 96 | TCF4 |  |  | 7166 | -0.041 | -0.6065 | No |
| 97 | LHFPL2 |  |  | 7179 | -0.041 | -0.6059 | No |
| 98 | NOTCH3 |  |  | 7196 | -0.042 | -0.6057 | No |
| 99 | EHD2 |  |  | 7220 | -0.043 | -0.6063 | No |
| 100 | CARMIL1 |  |  | 7234 | -0.043 | -0.6058 | No |
| 101 | SRGAP1 |  |  | 7250 | -0.043 | -0.6054 | No |
| 102 | PTCH1 |  |  | 7258 | -0.044 | -0.6042 | No |
| 103 | TMEM263 |  |  | 7263 | -0.044 | -0.6027 | No |
| 104 | ZNF217 |  |  | 7333 | -0.045 | -0.6081 | No |
| 105 | KDELR3 |  |  | 7339 | -0.046 | -0.6065 | No |
| 106 | SPATS2L |  |  | 7352 | -0.046 | -0.6058 | No |
| 107 | ACTN1 |  |  | 7366 | -0.046 | -0.6051 | No |
| 108 | RGL1 |  |  | 7385 | -0.047 | -0.6049 | No |
| 109 | SHROOM3 |  |  | 7472 | -0.050 | -0.6120 | No |
| 110 | RGS16 |  |  | 7474 | -0.050 | -0.6098 | No |
| 111 | COL5A3 |  |  | 7491 | -0.050 | -0.6093 | No |
| 112 | SCARA3 |  |  | 7514 | -0.051 | -0.6094 | No |
| 113 | PRRT2 |  |  | 7518 | -0.051 | -0.6074 | No |
| 114 | ID1 |  |  | 7519 | -0.051 | -0.6051 | No |
| 115 | CDKN1C |  |  | 7524 | -0.051 | -0.6032 | No |
| 116 | TSKU |  |  | 7606 | -0.053 | -0.6095 | No |
| 117 | CPVL |  |  | 7669 | -0.055 | -0.6138 | No |
| 118 | CNN2 |  |  | 7676 | -0.055 | -0.6119 | No |
| 119 | AFAP1 |  |  | 7747 | -0.058 | -0.6169 | No |
| 120 | FZD2 |  |  | 7754 | -0.058 | -0.6149 | No |
| 121 | APOD |  |  | 7757 | -0.058 | -0.6124 | No |
| 122 | CNPY4 |  |  | 7808 | -0.060 | -0.6152 | No |
| 123 | HES4 |  |  | 7819 | -0.060 | -0.6135 | No |
| 124 | LOXL2 |  |  | 7871 | -0.062 | -0.6162 | No |
| 125 | NUMBL |  |  | 7892 | -0.063 | -0.6156 | No |
| 126 | PLPP3 |  |  | 7911 | -0.063 | -0.6146 | No |
| 127 | TNFRSF12A |  |  | 7931 | -0.064 | -0.6138 | No |
| 128 | IL27RA |  |  | 8040 | -0.069 | -0.6224 | No |
| 129 | NBEA |  |  | 8075 | -0.070 | -0.6229 | Yes |
| 130 | RARRES3 |  |  | 8092 | -0.071 | -0.6214 | Yes |
| 131 | EGFR |  |  | 8094 | -0.071 | -0.6183 | Yes |
| 132 | SEC24D |  |  | 8096 | -0.071 | -0.6152 | Yes |
| 133 | FBN1 |  |  | 8107 | -0.072 | -0.6130 | Yes |
| 134 | ABCA5 |  |  | 8149 | -0.074 | -0.6141 | Yes |
| 135 | TRIM22 |  |  | 8154 | -0.075 | -0.6111 | Yes |
| 136 | ITGA5 |  |  | 8155 | -0.075 | -0.6078 | Yes |
| 137 | ODF2L |  |  | 8158 | -0.075 | -0.6046 | Yes |
| 138 | RNF24 |  |  | 8166 | -0.075 | -0.6019 | Yes |
| 139 | SH3BP5 |  |  | 8247 | -0.080 | -0.6070 | Yes |
| 140 | CXCL12 |  |  | 8251 | -0.080 | -0.6037 | Yes |
| 141 | IFI6 |  |  | 8257 | -0.080 | -0.6006 | Yes |
| 142 | NUCB2 |  |  | 8258 | -0.080 | -0.5969 | Yes |
| 143 | ZDHHC9 |  |  | 8264 | -0.081 | -0.5938 | Yes |
| 144 | RBFOX2 |  |  | 8282 | -0.082 | -0.5919 | Yes |
| 145 | ATP13A2 |  |  | 8295 | -0.083 | -0.5895 | Yes |
| 146 | ADAMTS9 |  |  | 8313 | -0.083 | -0.5875 | Yes |
| 147 | IFT20 |  |  | 8338 | -0.085 | -0.5863 | Yes |
| 148 | ZFHX4 |  |  | 8390 | -0.088 | -0.5878 | Yes |
| 149 | TSHZ2 |  |  | 8391 | -0.088 | -0.5838 | Yes |
| 150 | KCTD9 |  |  | 8406 | -0.089 | -0.5813 | Yes |
| 151 | SLC2A3 |  |  | 8419 | -0.090 | -0.5785 | Yes |
| 152 | ARHGAP29 |  |  | 8476 | -0.094 | -0.5803 | Yes |
| 153 | ARL4C |  |  | 8491 | -0.095 | -0.5775 | Yes |
| 154 | PDGFRA |  |  | 8503 | -0.096 | -0.5743 | Yes |
| 155 | AC009041.2 |  |  | 8526 | -0.099 | -0.5722 | Yes |
| 156 | TMEM59L |  |  | 8529 | -0.099 | -0.5680 | Yes |
| 157 | MEST |  |  | 8534 | -0.099 | -0.5639 | Yes |
| 158 | MIR222HG |  |  | 8537 | -0.099 | -0.5596 | Yes |
| 159 | BMP7 |  |  | 8549 | -0.100 | -0.5563 | Yes |
| 160 | ECE1 |  |  | 8556 | -0.100 | -0.5524 | Yes |
| 161 | ATP2B1 |  |  | 8584 | -0.102 | -0.5507 | Yes |
| 162 | PRICKLE1 |  |  | 8595 | -0.103 | -0.5471 | Yes |
| 163 | COL11A1 |  |  | 8598 | -0.103 | -0.5426 | Yes |
| 164 | GPATCH8 |  |  | 8606 | -0.104 | -0.5387 | Yes |
| 165 | COL8A1 |  |  | 8609 | -0.104 | -0.5342 | Yes |
| 166 | GLT8D1 |  |  | 8623 | -0.105 | -0.5308 | Yes |
| 167 | C1R |  |  | 8644 | -0.107 | -0.5282 | Yes |
| 168 | SCPEP1 |  |  | 8654 | -0.107 | -0.5243 | Yes |
| 169 | YIPF5 |  |  | 8663 | -0.108 | -0.5202 | Yes |
| 170 | PCOLCE |  |  | 8685 | -0.110 | -0.5175 | Yes |
| 171 | PLPPR5 |  |  | 8690 | -0.110 | -0.5130 | Yes |
| 172 | IL17RD |  |  | 8710 | -0.112 | -0.5099 | Yes |
| 173 | TSPAN13 |  |  | 8713 | -0.112 | -0.5051 | Yes |
| 174 | P3H1 |  |  | 8767 | -0.118 | -0.5055 | Yes |
| 175 | THBS1 |  |  | 8789 | -0.120 | -0.5023 | Yes |
| 176 | PLK2 |  |  | 8803 | -0.123 | -0.4981 | Yes |
| 177 | TPST1 |  |  | 8811 | -0.124 | -0.4933 | Yes |
| 178 | CPE |  |  | 8816 | -0.125 | -0.4880 | Yes |
| 179 | DTNA |  |  | 8828 | -0.126 | -0.4835 | Yes |
| 180 | DCLK1 |  |  | 8834 | -0.127 | -0.4783 | Yes |
| 181 | PERP |  |  | 8836 | -0.127 | -0.4726 | Yes |
| 182 | ASPH |  |  | 8843 | -0.128 | -0.4674 | Yes |
| 183 | TMEM158 |  |  | 8846 | -0.128 | -0.4618 | Yes |
| 184 | PLEKHH2 |  |  | 8850 | -0.129 | -0.4563 | Yes |
| 185 | MYOF |  |  | 8851 | -0.129 | -0.4504 | Yes |
| 186 | ANTXR1 |  |  | 8869 | -0.132 | -0.4463 | Yes |
| 187 | STIM2 |  |  | 8887 | -0.134 | -0.4420 | Yes |
| 188 | FRMD4A |  |  | 8896 | -0.135 | -0.4368 | Yes |
| 189 | BST2 |  |  | 8903 | -0.136 | -0.4313 | Yes |
| 190 | COL18A1 |  |  | 8909 | -0.137 | -0.4256 | Yes |
| 191 | MAP1A |  |  | 8919 | -0.137 | -0.4203 | Yes |
| 192 | TSPAN5 |  |  | 8951 | -0.142 | -0.4173 | Yes |
| 193 | COL21A1 |  |  | 8954 | -0.142 | -0.4110 | Yes |
| 194 | TNFRSF1A |  |  | 8962 | -0.143 | -0.4053 | Yes |
| 195 | CDH11 |  |  | 8983 | -0.146 | -0.4008 | Yes |
| 196 | FRMD4B |  |  | 9003 | -0.149 | -0.3961 | Yes |
| 197 | FGFR1 |  |  | 9034 | -0.155 | -0.3923 | Yes |
| 198 | PLIN3 |  |  | 9043 | -0.157 | -0.3861 | Yes |
| 199 | LRRC17 |  |  | 9058 | -0.159 | -0.3804 | Yes |
| 200 | TFPI |  |  | 9060 | -0.159 | -0.3732 | Yes |
| 201 | COL4A2 |  |  | 9069 | -0.161 | -0.3668 | Yes |
| 202 | CAVIN1 |  |  | 9071 | -0.161 | -0.3596 | Yes |
| 203 | SEZ6L2 |  |  | 9087 | -0.163 | -0.3538 | Yes |
| 204 | MRC2 |  |  | 9106 | -0.167 | -0.3482 | Yes |
| 205 | DNM1 |  |  | 9109 | -0.168 | -0.3408 | Yes |
| 206 | BOC |  |  | 9110 | -0.168 | -0.3331 | Yes |
| 207 | LDLR |  |  | 9117 | -0.170 | -0.3261 | Yes |
| 208 | PPIC |  |  | 9122 | -0.171 | -0.3188 | Yes |
| 209 | CDC42EP3 |  |  | 9126 | -0.171 | -0.3113 | Yes |
| 210 | SLC5A3 |  |  | 9147 | -0.177 | -0.3054 | Yes |
| 211 | COL5A1 |  |  | 9148 | -0.177 | -0.2974 | Yes |
| 212 | EMP1 |  |  | 9156 | -0.179 | -0.2900 | Yes |
| 213 | GALNT2 |  |  | 9171 | -0.182 | -0.2833 | Yes |
| 214 | AHNAK |  |  | 9183 | -0.185 | -0.2761 | Yes |
| 215 | SCRG1 |  |  | 9200 | -0.191 | -0.2691 | Yes |
| 216 | ATP6V0A1 |  |  | 9214 | -0.198 | -0.2615 | Yes |
| 217 | ID3 |  |  | 9252 | -0.214 | -0.2558 | Yes |
| 218 | COL4A1 |  |  | 9284 | -0.226 | -0.2489 | Yes |
| 219 | IGFBP7 |  |  | 9295 | -0.229 | -0.2396 | Yes |
| 220 | CDKN1A |  |  | 9301 | -0.230 | -0.2297 | Yes |
| 221 | COL5A2 |  |  | 9316 | -0.244 | -0.2202 | Yes |
| 222 | LRP1 |  |  | 9338 | -0.259 | -0.2107 | Yes |
| 223 | KCNE4 |  |  | 9344 | -0.264 | -0.1992 | Yes |
| 224 | NRP2 |  |  | 9357 | -0.281 | -0.1877 | Yes |
| 225 | SPARCL1 |  |  | 9360 | -0.288 | -0.1749 | Yes |
| 226 | FN1 |  |  | 9364 | -0.294 | -0.1618 | Yes |
| 227 | SULF2 |  |  | 9376 | -0.313 | -0.1488 | Yes |
| 228 | VCAN |  |  | 9396 | -0.364 | -0.1343 | Yes |
| 229 | CTGF |  |  | 9400 | -0.375 | -0.1176 | Yes |
| 230 | DKK3 |  |  | 9411 | -0.414 | -0.0999 | Yes |
| 231 | MEG3 |  |  | 9419 | -0.461 | -0.0798 | Yes |
| 232 | IGFBP3 |  |  | 9424 | -0.502 | -0.0574 | Yes |
| 233 | CLU |  |  | 9430 | -0.547 | -0.0331 | Yes |
| 234 | POSTN |  |  | 9437 | -0.750 | 0.0003 | Yes |
Table: GSEA details [plain text format]

  

Fig 2: DOROSZKO\_U3054MG\_CLUSTER\_2: Random ES distribution      
 Gene set null distribution of ES for **DOROSZKO\_U3054MG\_CLUSTER\_2**
